# DNA methylation markers for disease progression and survival in Barrett’s Esophagus

**DOI:** 10.1186/1756-8935-6-S1-P81

**Published:** 2013-03-18

**Authors:** Marc Taenzer, Michael Quante, Magdalena Liebl, Roland M Schmid

**Affiliations:** 1 Klinkum r.d. Isar, Technische Universität München, Munich, Bavaria, Germany

## 

Esophageal adenocarcinoma (EAC), with its precursor lesion Barrett’s esophagus (BE), accounts for 2% of all cancer-related deaths and has a very poor prognosis with a median survival of less than one year. In addition, the prevalence of BE is rapidly rising in the Western World, resulting in a large number of individuals “at risk” for this disease. However, a major limitation for defining this risk population is the absence of reliable biomarkers, which also affects the development of surveillance strategies and chemoprevention therapies for BE and EAC. The development of BE has been thought to arise largely because of chronic esophageal inflammation and the subsequent the expansion of some sort of an altered stem cell population due to alteration of stromal niche factors. This also leads to epigenetic alterations, which are common in cancer initiation and progression, such as changes in DNA methylation. Many genes show great promise as specific DNA methylation biomarkers, which offer several advantages, as they are easy accessible in body fluids such as blood, sputum, or urine and the DNA containing the methylation information can be isolated from formalin fixed paraffin embedded (FFPE) tissue as well; the positive methylation signal can also be detected in the presence of huge amounts of material from normal cells giving a negative signal. We measured two DNA methylation markers, DKK1 (a WNT antagonist, known to be methylated in colorectal and gastric cancer which has a function tumor cell niche formation) and TFAP2E (a novel biomarker for nonresponse towards 5FU in colon cancer) in a retrospective cohort of patients with EAC collected at the Institute of Pathology at the TU Munich (FFPE material), using Methylspecific-HRM-PCR followed by pyrosequencing.

## Results

In a cohort of 60 EAC patients (primary resections), DKK1 methylation was associated with overall survival (ROC analysis, AUC 0.69, 95% CI, p < 0,001) as well as disease free survival (AUC 0.63, 95% CI, p < 0,01). In another cohort of 55 patients who received neoadjuvant chemotherapy (5FU, cisplatin, folinic acid) TFAP2E methylation was found also to be associated with overall survival (AUC 0.64, 95% CI, p < 0,05) but less so if patients who did not receive 5FU (taxol, cisplatin) were included (AUC 0.62,95% CI, p < 0,05).

